# Conspicuity of Peripheral Zone Prostate Cancer on Computed Diffusion-Weighted Imaging: Comparison of cDWI_1500_, cDWI_2000_, and cDWI_3000_

**DOI:** 10.1155/2014/768291

**Published:** 2014-12-01

**Authors:** Metin Vural, Gökhan Ertaş, Aslıhan Onay, Ömer Acar, Tarık Esen, Yeşim Sağlıcan, Hale Pınar Zengingönül, Sergin Akpek

**Affiliations:** ^1^Department of Radiology, VKF American Hospital, Nisantası, 34365 Istanbul, Turkey; ^2^Department of Biomedical Engineering, Yeditepe University, Atasehir, 34755 Istanbul, Turkey; ^3^School of Medicine, Koç University, Topkapı, 34104 Istanbul, Turkey; ^4^Department of Urology, VKF American Hospital, Nisantası, 34365 Istanbul, Turkey; ^5^Department of Pathology, School of Medicine, Acibadem University, Atasehir, 34752 Istanbul, Turkey; ^6^Department of Biomedical Engineering, Namık Kemal University, Merkez, 59000 Tekirdağ, Turkey

## Abstract

*Introduction and Objective*. Disadvantages associated with direct high* b*-value measurements may be avoided with use of computed diffusion-weighted imaging (cDWI). The purpose of this study is to assess the diagnostic performance of cDWI image sets calculated for high* b*-values of 1500, 2000, and 3000 s/mm^2^. * Materials and Methods*. Twenty-eight patients who underwent multiparametric MRI of the prostate and radical prostatectomy consecutively were enrolled in this retrospective study. Using a software developed at our institute, cDWI_1500_, cDWI_2000_, and cDWI_3000_ image sets were generated by fitting a monoexponential model. Index lesions on cDWI image sets were scored by two radiologists in consensus considering lesion conspicuity, suppression of background prostate tissue, distortion, image set preferability, and contrast ratio measurements were performed.* Results*. Lesion detection rates are the same for computed* b*-values of 2000 and 3000 s/mm^2^ and are better than* b*-values of 1500 s/mm^2^. Best lesion conspicuity and best background prostate tissue suppression are provided by cDWI_3000_ image set. cDWI_2000_ image set provides the best zonal anatomical delineation and less distortion and was chosen as the most preferred image set. Average contrast ratio measured on these image sets shows almost a linear relation with the* b*-values. Conclusion. cDWI_2000_ image set with similar conspicuity and the same lesion detection rate, but better zonal anatomical delineation, and less distortion, was chosen as the preferable image set.

## 1. Introduction

Prostate cancer, the most frequently diagnosed cancer in men, aside from skin cancer, is a major public health issue in the world today [1]. The development of minimally invasive procedures such as imaging-guided brachytherapy, intensity-modulated radiation therapy, high-intensity focused ultrasound, and cryotherapy in prostate cancer treatment has increased interest in improving the detection and localization of prostate cancer [2].

Multiparametric prostate magnetic resonance imaging (mp-MRI) has revealed an increased level of spatial, anatomic, and functional information and has shown promise for improved detection and characterization of prostate cancer [3]. Diffusion-weighted MRI (DW-MRI) is a useful imaging technique and a powerful component of mp-MRI of the prostate. DW-MRI of the abdomen has been significantly improved in the past few years as well. Technical innovations such as the use of fast gradients, multichannel coils, and parallel imaging have resulted in reduced acquisition times for DW-MRI, thus minimizing some of its limitations of motion, susceptibility, and chemical-shift artifacts [4].

In DW-MRI,* b*-value identifies the sensitivity to diffusion and adjusts the strength and duration of the diffusion gradients as well as the time interval between the paired gradients. There is a considerable debate regarding appropriate* b*-values for DW-MRI of the prostate. Normal prostate tissue, especially in the TZ, may reveal high signal intensity on diffusion-weighted MR images and low ADC, thus mimicking a tumor [3]. Recent studies have reported positive results using a* b*-value of >1000 s/mm^2^ [5–7] to overcome this problem. However, even though higher* b*-value images are clinically desirable, obtaining images with a high* b*-value by direct measurement is challenging. Such images have an inherently low signal-to-noise ratio (SNR) and are prone to increased susceptibility artefact and severe eddy current distortions from the large diffusion-sensitizing gradients used. Kitajima et al. [8] showed that as the* b*-value increased from 1000 to 2000 s/mm^2^, the mean SNR of prostate cancer decreased by 21.6%.

Computed DW imaging (cDWI) is a mathematical technique which calculates a high* b*-value image from DW-MR images acquired with at least two different lower* b*-values. In this way, disadvantages associated with direct high* b*-value measurements such as poor SNR and image distortion may be avoided. It has been recently reported that computed high* b*-value diffusion-weighted images of the prostate improves tumor separability and image quality [9, 10].

In this study, we have carried out retrospective work to directly compare different generated DW images (cDWI_1500_, cDWI_2000_, and cDWI_3000_ s/mm^2^) on lesion conspicuity and image quality using whole mount-section histopathological examination as the reference standard.

## 2. Materials and Methods

### 2.1. Patient Population and Imaging Protocol

Thirty-eight patients who underwent multiparametric MRI of the prostate and radical prostatectomy consecutively between December 2012 and April 2014 were enrolled in the study group. The median interval between MRI examinations and radical prostatectomy was 66 days (between 6 days to 6.5 months). The patients ranged in age from 46 to 71 years (mean, 60 years) and had serum PSA levels ranging from 3.7 to 40 ng/mL (mean, 9.7 ng/mL). The institutional and research committee waived informed consent and approved this retrospective study.

During histopathological analysis, prostatectomy specimens were fixed in 10% buffered neutral formalin for a minimum of 24 hours. Surgical margins were painted with different colors of ink to allow for unequivocal identification of left and right sides. Prostate tissue was serially sectioned at 3-4 mm thickness by knife perpendicular to the long axis of the prostate (from apex to base). Each of the slices was sequentially submitted in total for routine tissue processing and as whole mount sectioning. Routine sections were stained with hematoxylene and eosin. Whole mount mega and standard slides were reviewed by a pathologist (Y.S.) with 10 years of experience in uropathology.

Index lesions, considered to be the largest lesion with a high Gleason score for each patient, were recorded on a standardized diagram of the prostate divided into 16 sectors (ten peripheral zones and six transition zones) and by anatomical landmarks (namely, the prostatic capsule, the pseudocapsule, the urethra, and the ejaculatory ducts) by the pathologist and study coordinator radiologist (S.A.). The mean maximal size of tumor in histopathological specimens was 1.3 cm (ranging from 0.4 to 2.4 cm).

Diffusion-weighted MR images were acquired using a 3T MR scanner (Magnetom Skyra, Siemens Medical Solutions, Erlangen, Germany) with spine and sixteen-channel phased array coils as a part of the multiparametric prostate MRI protocol. No endorectal coil was used. Peristalsis was suppressed with intramuscular administration of 20 mg of butylscopolamine (Buscopan; Boehringer, Germany). No bowel preparation was performed. A free-breathing single-shot echo-planar imaging sequence was used with the following parameters: Repetition/Echo Time (TR/TE) = 4000/101 ms; matrix size = 192 × 154; field of view = 260 × 260 mm; slice thickness/gap = 3.6 mm/0.3 mm; 22 axial slices;* b*-values = 0, 50, 100, 200, 400, 600, and 800 s/mm^2^ with nine excitations; and an overall acquisition time of 5 mins. The maximum gradient amplitude per axis was 45 mT/m and the maximum slew rate was 200 T/m/s. All images were anonymized and transferred to a workstation in DICOM format with 16-bit greyscale intensity for subsequent analysis.

### 2.2. Calculation of High* b*-Value Diffusion-Weighted Images

For a volume imaged using DW-MRI, apparent diffusion coefficient (ADC) can be calculated on voxel basis using a monoexponential model. Let *S*_*n*_(*x*, *y*, *z*) be the signal intensity value of the voxel with coordinates (*x*, *y*, *z*) in the volume imaged using diffusion-weighted imaging with *N* different* b*-values (*n* = 1,2,…, *N*). The relation between apparent diffusion coefficients for a voxel located at (*x*, *y*, *z*) can be given by

(1) $$\begin{array}{c} S_{n}\left(x,y,z\right)=S_{1}\left(x,y,z\right)\exp^{-(b_{n}-b_{1})\text{ADC}}. \end{array}$$

An ADC estimate can be computed from diffusion-weighted image sets acquired with at least two different* b*-values using

(2) $$\begin{array}{c} \overset{-}{\text{ADC}}=\underset{\text{ADC}}{\arg}\max P\left(S\;|\;\text{ADC}\right), \end{array}$$

where *P*  (*S*_*n*_∣ADC) ~ *N*(*S*_1_exp⁡^−(*b*_*n*_ − *b*_1_)ADC^, *σ* ^2^).

The ADC estimate can next be used to calculate the signal intensity of the voxel *S*_*c*_(*x*, *y*, *z*) for an ultrahigh* b*-value *b*_*c*_ using

(3) $$\begin{array}{c} S_{c}\left(x,y,z\right)=S_{1}\left(x,y,z\right)\exp^{-(b_{c}-b_{1})\overset{-}{\text{ADC}}}. \end{array}$$

In this study, ADC estimations and calculations were performed numerically using “DWMRI Mapper” software developed at our institute written in MATLAB 8.1 (The Mathworks Inc., Natick, MA). This software reads the diffusion-weighted MR image sets in DICOM format and performs a nonlinear least square fitting based on Trust-Region fitting algorithm voxel-by-voxel basis to determine the ADC estimates. The estimates are next used to automatically calculate the signal intensity values of the voxels for* b*-values of 1500, 2000, and 3000 s/mm^2^. The software stores the signal intensity values calculated in DICOM image files in unique sets according to the* b*-value used, namely, cDWI_1500_, cDWI_2000_, and cDWI_3000_. These image sets can be transferred to workstations at the clinic for use in evaluations.

### 2.3. Qualitative Evaluation and Scoring

All MR images of the patients enrolled in this study were evaluated in consensus by two radiologists (M.V., A.O., 14 and 5 years of experience in abdominal MRI and 4 and 2 years in prostate MRI, resp.) using a MRI CAD system (DynaCad; Invivo Birmingham, MI). The radiologists knew that all patients had undergone radical prostatectomy; however, they were blinded to all patient's identifiers, clinical presentation, and histopathologic and imaging parameters. While reviewing each diffusion-weighted image set (cDWI_1500_, cDWI_2000_, and cDWI_3000_) in random order, radiologists identified the location of the index lesion based on its increased signal intensity relative to the background prostate parenchyma. These findings were then compared to lesions previously diagrammed by the uropathologist and the study coordinator. Surgical specimens often shrink after formaldehyde fixation so alignment of MR and whole mount-section histopathology findings was quite difficult. Therefore, a tumor detected on a computed diffusion-weighted image was considered as the matched lesion if any part of the tumor was present within the same area on the histopathologic diagram. Only lesions recorded at the correct location determined by the study coordinators were accepted as true-positive. The two radiologists also subjectively scored the three randomized image sets on five-point Likert-like scale (where a score of 5 denotes highest image quality whilst a score of 1 denotes very poor image quality) considering criteria adopted from Rosenkrantz et al. [11]: lesion conspicuity, image distortion, background suppression, and image set preferability. At least two weeks interval was given between each image set assessment.

After scoring, using DynaCad Software (DynaCad; Invivo Birmingham, MI), the study coordinator (S.A.) manually placed a pair of circular regions of interest (ROIs) on generated DW images (one for the index lesion and one for the healthy counterpart with reference to the whole mount-section histopathology slides). The average area of the ROIs defined was 10 ± 2 mm^2^. While delineating the ROIs, in order to reduce any error in contrast to noise ratio, great care was taken to exclude the urethra using high precision, T2-weighted images as a reference. The ROIs defined were then copied onto the cDWI images. For each ROI pair, the average signal intensity for the index lesion, SI_lesion_, and the average signal intensity for the healthy counterpart, SI_healthy_, were noted and then used to measure the contrast ratio given by

(4) $$\begin{array}{c} \text{CR}=\frac{{\text{SI}}_{\text{lesion}}-{\text{SI}}_{\text{healthy}}}{{\text{SI}}_{\text{lesion}}+{\text{SI}}_{\text{healthy}}}. \end{array}$$

### 2.4. Statistical Analysis

All statistical analyses were performed using SPSS 15 (SPSS Inc., USA). Contrast ratio measurements and scorings from cDWI_1500_, cDWI_2000_, and cDWI_3000_ were compared using the Tukey-Kremer test. A two-tailed *P* value < 0.05 was considered significant.

## 3. Results

**Figure 1:**
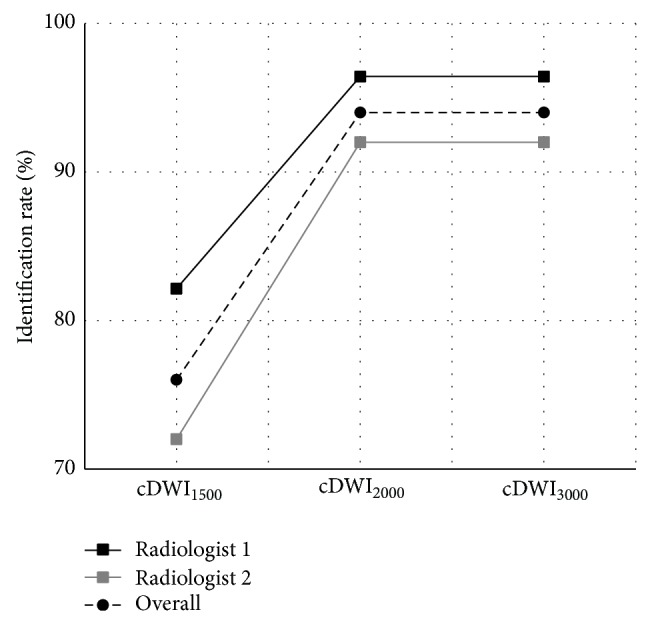
Lesion identification rates.

Number of index lesions identified by the two radiologists (radiologist 1: M.V and radiologist 2: A.O) on the cDWI image sets of the twenty-five patients and the corresponding lesion identification rates are as given in Table 1. Just for a single patient, the lesion is unnoticeable on any cDWI image set. In the rest of the patients, index lesions are at least identified on one image set. Overall lesion identification rates are 76%, 94%, and 94% for cDWI_1500_, cDWI_2000_, and cDWI_3000_ image sets, respectively. This finding shows that lesion identification performance improves as the* b*-value for the computed diffusion-weighted image set increases but it does not improve more for* b*-values higher than 2000 s/mm^2^ as illustrated in Figure 1.

**Figure 2:**
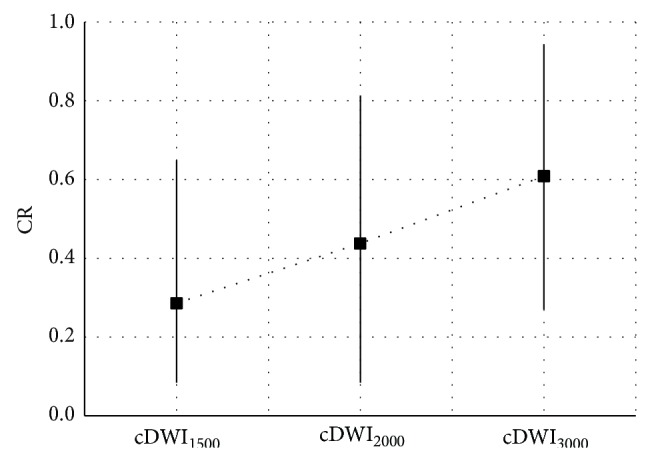
Contrast ratios measured.

Figure 2 shows the results of contrast ratio measurements. Average contrast ratios measured on cDWI_1500_, cDWI_2000_, and cDWI_3000_ image sets are 0.29 ± 0.13, 0.43 ± 0.18, and 0.60 ± 0.20, respectively, showing almost a linear relation between computed image sets. On average, contrast ratio is at its maximum when cDWI_3000_ image set is considered; however, it decreases on cDWI_2000_ and cDWI_1500_ image sets. Significant differences in contrast ratio are obtained between cDWI_1500_ and cDWI_2000_ image sets (*P* = 0.015) and and cDWI_3000_ image sets (*P* = 0.002). However, difference in contrast ratio is most significant between DWI_1500_ and cDWI_3000_ image sets (*P* < 0.001).

**Figure 3:**
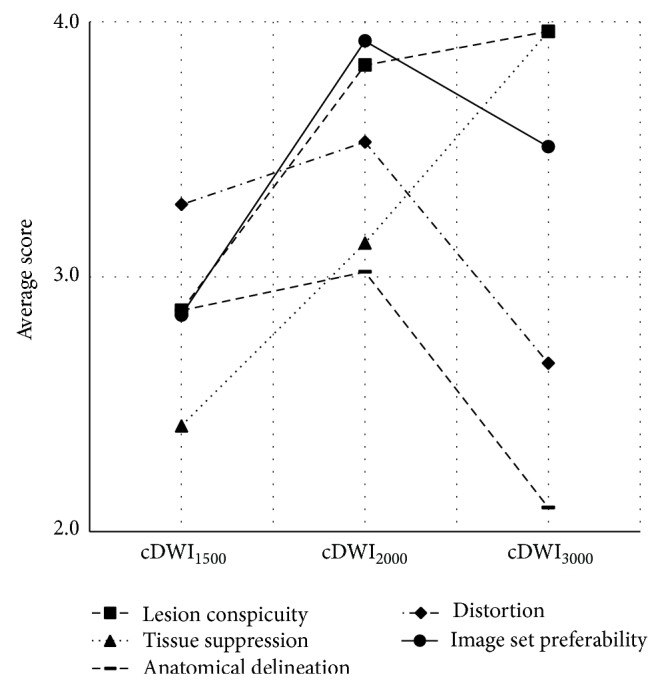
Average score plots.

**Figure 4:**
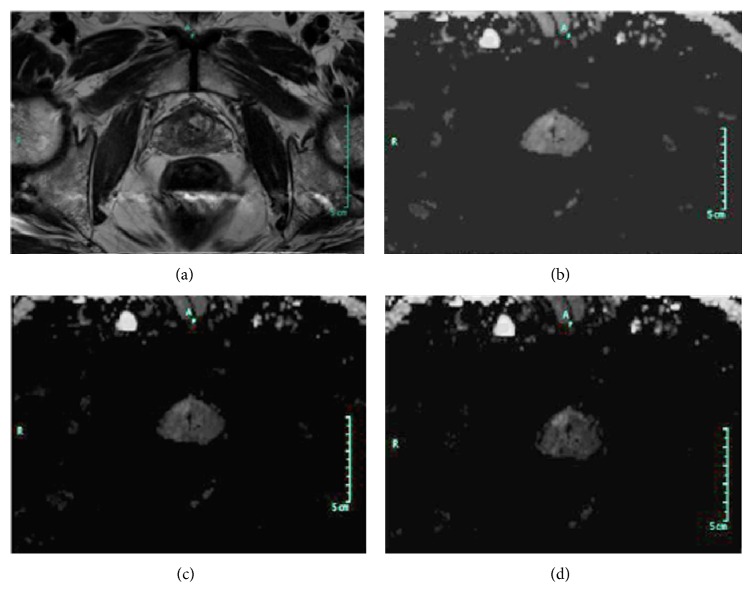
Prostate cancer in a 65-year-old man with a PSA level of 10.5 ng/mL and a Gleason score of 5 + 4. (a) Transverse T2-weighted turbo-spin echo MR image shows the index lesion on midgland, right transitional zone. (b) On cDWI_1500_ image set anteriorly located lesion was barely seen because of inadequate background suppression. (c, d) On cDWI_2000_ and cDWI_3000_ image sets index lesion was more conspicuous. Also a tiny biopsy proven tumoral focus was identified in the right peripheral zone on cDWI_2000_ and cDWI_3000_ image sets.

Average of Likert-like scores given in consensus by the two radiologists is seen in Table 2. Between cDWI_2000_ and cDWI_3000_ image sets, significant difference in scores exists for background prostate tissue suppression, zonal anatomical delineation, and distortion (for all *P* < 0.001); however, difference is insignificant in scores for lesion conspicuity (*P* = 0.72) and image set preferability (*P* = 0.28). Between cDWI_1500_ and cDWI_2000_ image sets, significant difference in scores exists for lesion conspicuity, background prostate tissue suppression, and image set preferability (for all *P* < 0.001); however, difference is insignificant in scores for distortion (*P* = 0.43) and zonal anatomical delineation (*P* = 0.59). Plots of average scores determined for the image sets are presented in Figure 3. Lesion conspicuity and background prostate tissue suppression get highest scores for cDWI_3000_ image set (Figure 4). These scores increase as the* b*-value of the computed diffusion-weighted image set increases. A linear correlation is noticeable between the background prostate tissue suppression scores and the computed image sets. In addition to these, highest scores for zonal anatomical delineation and distortion are given for cDWI_2000_ image set. These scores are at their lowest value for cDWI_3000_ image set. With respect to the preferability of image set for use in prostate cancer evaluations, cDWI_2000_ image set takes the highest score from radiologists. These results show that among cDWI_1500_, cDWI_2000_, and cDWI_3000_ image sets, cDWI_2000_ image set provides the best performance facilitating the conspicuity of prostate cancer on computed diffusion-weighted imaging.

## 4. Discussion

DWI is an essential component of mp-MRI, enabling qualitative and quantitative assessments of prostate cancer aggressiveness. The signal in DWI decays as a function of the amount of incoherent motion present in the tissue and a diffusion weighting parameter known as* b*-value. A greater* b*-value indicates a more severe phase dispersion of water molecules and a more reduced signal under the effect of gradient pulse on DW imaging [4]. Most DW-MRI examinations in the body utilize* b*-values between 0 and 10000 s/mm^2^. Considerations for image quality and signal-to-noise ratio restrict the use of ultrahigh* b*-values for imaging (e.g.,* b-*value of 2000 s/mm^2^) [8]. In a 2012 guideline, the European Society of Urogenital Radiology recommended* b*-values of 0, 100, 500, and 800–1000 s/mm^2^, for optimal DW images [3]. Within this range of* b*-values, the peripheral zone of the prostate gland frequently still appears hyperintense on the DW images which is due to relatively long T2-relaxation time of the glandular tissue. This can be referred to as a “T2 shine through effect,” which can confound disease detection. For this reason, reviewing the ADC maps of the prostate is more useful for disease detection in the peripheral prostate gland. Since benign and normal tissues tend to show greater signal attenuation at high* b*-values compared to cellular tumors, the use of high* b*-value DW-MRI has been recognized as a method to increase the radiological conspicuity.

The potential of sufficiently high* b*-value DW images has been reported to improve the detection rate of different malignant tissue types [10, 12, 13]. Recent studies have compared DW images obtained using high* b*-values for prostate cancer detection and reached conflicting results; Katahira et al. [6], Rosenkrantz et al. [9], Ueno et al. [7], and Ohgiya et al. [14] reported the advantage of a* b*-value of 2000 s/mm^2^ compared to a* b*-value of 1000 s/mm^2^, whereas Kim et al. [15] reported no benefit from the greater* b*-values. According to Metens et al. [5], native DW images with a* b*-value of 2000 s/mm^2^ have better contrast-to-noise ratio in comparison with a* b*-value of 1000 s/mm^2^ but lower than those with a* b*-value of 1500 s/mm^2^.

Maas et al. demonstrated that cDWI is a method capable of obtaining high* b*-value images, which avoids the technical challenges of actually measuring them [16]. Rosenkratz et al. [11] compared acquired and generated* b*-1500 s/mm^2^ images and stated that DW images at* b*-values greater than 1000 s/mm^2^ might be routinely incorporated into mp-MRI protocols. Glaister et al. [10] reported a quantitative investigative analysis of the improvement in tumor differentiation in the prostate gland and emphasized diagnostic performance of computed DWI with a* b*-value around 3000 s/mm^2^.

There are several limitations of this study. First, it is vulnerable to the inherent disadvantages of its retrospective design. Second, a degree of selection bias has been introduced by including patients with prostate cancer who underwent radical prostatectomy. Third, by the preparation of the whole mount specimen, tissue may shrink; therefore, MR images of the prostate are not necessarily perpendicular to the prostatic urethra, making a comparison of pathologic and MR imaging findings potentially problematic. Fourth, computed diffusion-weighted images have been generated by using a monoexponential model. Kimura and Machii [17] demonstrate that the monoexponential model may fit for computed DWI theory, but some other studies have demonstrated the potential of biexponential models for depicting prostate cancer [18, 19]. Finally, in this study rather than all tumor foci, the index lesions have been evaluated. Liu et al. [20] report that all metastatic sites in a single patient derives from a single monoclonal precursor cell, indicating that despite the multifocality of prostate cancer, a single tumor focus (i.e., index lesion) is responsible for tumor progression and death. This suggests that emerging focal therapy techniques based on index lesion may achieve adequate control of this multifocal disease.

In conclusion, computed DWI is a promising technique in prostate cancer detection that has the potential to eliminate hardware limitations of MR scanners such as lower SNR and image quality. Although the number of patients enrolled in this study is less than ideal, the results indicate that cDWI_3000_ image set is superior to cDWI_2000_ and cDWI_1500_ image sets in terms of conspicuity of index lesions. However, cDWI_2000_ image set provides similar conspicuity but better zonal anatomical delineation and distortion suppression and is the preferable image set for use in localizing cancer in prostate gland. Further studies with larger groups and with more sophisticated computation methods that employ biexponential models or stretched exponential models should determine the optimal computed* b*-value.

## Figures and Tables

**Table 1:** Number of index lesions identified and identification rates.

	cDWI_1500_	cDWI_2000_	cDWI_3000_
Radiologist 1	20 (80%)	24 (96%)	24 (96%)
Radiologist 2	18 (72%)	23 (92%)	23 (92%)
Overall	19 (76%)	23.5 (94%)	23.5 (94%)

**Table 2:** Average of scores given in consensus.

	cDWI_1500_	cDWI_2000_	cDWI_3000_
Lesion conspicuity	2.66 ± 1.52	3.72 ± 1.02	**3.91 ± 1.00**
Background prostate tissue suppression	2.31 ± 1.27	3.11 ± 0.87	**3.89 ± 0.79**
Zonal anatomical delineation	2.83 ± 1.45	**3.04 ± 0.86**	2.15 ± 0.72
Distortion	3.26 ± 1.65	**3.55 ± 0.93**	2.72 ± 0.71
Image set preferability	2.70 ± 1.41	**3.89 ± 0.91**	3.55 ± 0.83
